# Comment on Alley, S.J., et al. As the Pandemic Progresses, How Does Willingness to Vaccinate against COVID-19 Evolve? *Int. J. Environ. Res. Public Health* 2021, *18*, 797

**DOI:** 10.3390/ijerph18062809

**Published:** 2021-03-10

**Authors:** Jakob Weitzer, Manfred D. Laubichler, Brenda M. Birmann, Martin Bertau, Lukas Zenk, Guido Caniglia, Carlo C. Jäger, Gerald Steiner, Eva Schernhammer

**Affiliations:** 1 Department of Epidemiology, Medical University of Vienna, 1090 Vienna, Austria; weitzer.jakob@gmail.com; 2 Complexity Science Hub, Vienna, 1080 Vienna, Austria; manfred.laubichler@asu.edu (M.D.L.); carlo.jaeger@globalclimateforum.org (C.C.J.); 3 School of Complex Adaptive Systems, Arizona State University, Tempe, AZ 85281, USA; 4 Santa Fe Institute, Santa Fe, NM 87501, USA; 5 Global Climate Forum, 10178 Berlin, Germany; 6 Channing Division of Network Medicine, Department of Medicine, Brigham and Women’s Hospital and Harvard Medical School, Boston, MA 02115, USA; brenda.birmann@channing.harvard.edu; 7 Institute of Chemical Technology, Freiberg University of Mining and Technology, 09599 Freiberg, Germany; martin.bertau@chemie.tu-freiberg.de; 8 Department for Knowledge and Communication Management, Danube University, 3500 Krems a. d. Donau, Austria; lukas.zenk@donau-uni.ac.at; 9 Konrad Lorenz Institute for Evolution and Cognition Research, 3400 Klosterneuburg, Austria; guido.caniglia@kli.ac.at

We would like to extend on the article by Alley et al. [1], providing additional data in response to their message of how the willingness to vaccinate is evolving, the need for promoting a COVID-19 vaccine among certain subgroups of the population, and communication strategies.

As the COVID-19 pandemic continues to surge worldwide, recent approvals of COVID-19 vaccines raise hope for a light at the end of the long and dark tunnel. Yet COVID-19 vaccination programs can affect meaningful resolution only with sufficient participation rates to achieve herd immunity (assumed to occur when about 60% of the population becomes immune and higher, if initially only adults will receive the vaccine [2]). A global survey conducted 16–20 June 2020 revealed promising levels of potential vaccine acceptance (55–89%) assuming that an available COVID-19 vaccine had been shown to be safe and effective [3].

However, the public’s attitudes towards COVID-19 have continued to evolve. Acceptance levels for restrictions and trust in authorities have diminished, fueled in part by political polarization [4], and hesitancy towards a COVID-19 vaccine has increased [1,5]. Similar trends are also evidenced in a survey of the Austrian population (26 November through 3 December 2020). Of 1007 population-representative participants, 53.8% declared that they did not trust the Austrian government with respect to a safe vaccine (e.g., rather not, or not at all), and merely 36.1% indicated that they would be likely or very likely to get vaccinated once a COVID-19 vaccine is available in Austria (with 22.8% undecided). The unwillingness to vaccinate was higher among those in favor of political opposition parties (47.2%) and those who did not vote in the last national elections (51.6%), and lowest among participants favoring the parties in power (32.2%).

In addition, willingness to adhere to Coronavirus protection measures changed substantially between March and April 2020 (first lockdown) and June and October 2020; total approval of protection measures dropped from 59 to 47%, partial approval increased from 31 to 39%, and disapproval increased from 10 to 14%. Further, participants who indicated that they did not adhere to social distancing (13.2% of the sample) more frequently reported an unwillingness (rather or very unlikely) to vaccinate (68.9%) compared to participants who indicated overall adherence to social distancing (36.9% rather or very unlikely to vaccinate).

These numbers reflect a lack of trust in the political system [6]. But there is hope for changing these trends. In our survey, 80.7% wanted to see the results of an independent scientific evaluation of the vaccine before getting vaccinated, suggesting that an independent and de-politicized evaluation of COVID-19 vaccines and transparent communication of results by trusted scientists and community stakeholders can increase the willingness to be vaccinated. In the upcoming months, it will thus be essential to invest in transparent and effective ways to communicate results and achievements to the broader public.

## Data Availability

The data presented in this study are available from the corresponding authors, upon reasonable request.
